# Associations between chronotype and psychiatric symptoms across the adult lifespan

**DOI:** 10.1038/s41398-025-03782-w

**Published:** 2025-12-05

**Authors:** John Axelsson, Leonie JT Balter

**Affiliations:** 1 Department of Clinical Neuroscience, Karolinska Institutet, Stockholm, Sweden; 2 Department of Psychology, Stockholm University, Stockholm, Sweden; 3 Department of Psychiatry, Radboud University Nijmegen Medical Centre, Nijmegen, the Netherlands; 4 Donders Institute for Brain, Cognition and Behaviour, Radboud University Nijmegen, Nijmegen, the Netherlands

**Keywords:** Human behaviour, Psychiatric disorders

## Abstract

Chronotype, the individual propensity towards morningness or eveningness, is a transdiagnostic factor implicated in mental health. How chronotype covaries with distinct psychiatric symptoms across the adult lifespan remains underinvestigated. In this cross-sectional study, 428 participants (18–70 years, evenly distributed across five age groups; 282 women, 143 men, other gender identity) completed the Reduced Morningness-Eveningness Questionnaire to approximate chronotype, and 11 validated scales assessing common psychiatric symptoms. Regression analyses and generalized additive models (GAMs) were used to examine linear and nonlinear relationships between chronotype, age, and psychiatric symptoms. Eveningness was associated with (in decreasing order of strength): emotion dysregulation, attention-deficit/hyperactivity disorder (ADHD), depression, autism, emotional instability, generalized anxiety, social anxiety, and impulsivity symptoms. Morningness was associated with mania symptoms. No significant associations were found between chronotype and delusional ideation or obsessive compulsive disorder (OCD) symptoms. Psychiatric symptom severity was generally lower with increasing age, particularly for emotional instability, emotion dysregulation, and ADHD. Nonetheless, certain symptoms persisted across age in eveningness (depression, generalized anxiety, OCD, ADHD), while other symptoms were lower (mania) or higher (social anxiety and delusional ideation) with eveningness at older ages. Except for mania symptoms, morningness in older age was not associated with higher symptom burden than eveningness for any other psychiatric domains. These results are consistent with the idea that morningness may be protective, being associated with lower psychiatric symptom levels in both younger and older age groups. In contrast, eveningness was associated with higher levels of autism, social anxiety and delusional ideation symptoms in older age.

## Introduction

The initial appearance of different psychiatric disorders occurs at various life stages [1–3]. For instance, autism spectrum disorders (ASD), attention deficit hyperactivity disorder (ADHD), and social anxiety are often diagnosed before adolescence, whereas anxiety disorders, eating disorders, and obsessive compulsive disorder (OCD) diagnoses tend to peak in late adolescence. Early adulthood is related to a rise in diagnoses of personality disorders, schizophrenia, and bipolar disorder, and major depressive disorder is more frequently diagnosed in adulthood [1–3]. The prevalence of psychiatric disorders among older adults is less well-documented compared to younger adults. In a nationally representative U.S. sample of older adults aged 55–85+, anxiety and personality disorders were more common than mood and substance use disorders, with an overall decline in psychiatric disorders observed from ages 55 to 85+ [4]. Several factors have been proposed to explain why psychiatric disorders are less prevalent in older than in younger adults, including reduced societal pressures and stressors after retirement, smaller but more intimate social networks with more emotionally close relationships, as well as changes in diagnostic criteria, public awareness, and a larger focus on diagnosing younger individuals [5–8]. It has, for example, been estimated that more than 90% of people with autism over the age of 50 are undiagnosed [9]. Given that psychiatric disorders are associated with adverse physical health outcomes, reduced quality of life, higher rates of age-related diseases, and shorter life expectancy [10, 11], identifying which psychiatric symptoms are more prevalent or problematic at specific ages can help tailor support systems to the changing needs of individuals as they age.

Chronotype, which represents the behavioral manifestation of underlying circadian rhythms [12], is associated with mental health. An earlier diurnal preference, i.e., a morning chronotype or “morningness”, is considered a protective factor against major depressive disorder, and phase advancing evening chronotypes leads to improvements in symptoms of depression and stress [13, 14]. In contrast, the evening chronotype, or “eveningness”, is linked to the severity of mood disorder symptoms, in both clinical and non-clinical populations [14–16]. A growing body of evidence suggests that eveningness is a phenotype that cuts across multiple psychiatric issues [17], although the full spectrum of the relationship between psychiatric symptoms and chronotype is not fully characterized, particularly in older age groups. Although chronotype has a genetic basis [18, 19] and is often considered a stable state classification [20], it changes across the lifespan. Eveningness is more prevalent in early adulthood, with a shift towards morningness as individuals age [21, 22]. Morningness tends to be more stable with age than eveningness [23]. Chronotype and mental health both change across the lifespan, and there are hints that the relationship between chronotype and depression may follow a nonlinear pattern [24]. These observations motivate an explicit test of whether, and if so, how the associations between chronotype and psychiatric symptoms vary across adulthood.

The relationship between chronotype and mental health extends beyond clinical psychiatric disorders, also manifesting in individuals who do not meet diagnostic criteria for specific psychiatric conditions (e.g., [15, 25]). To capture this broader spectrum of mental health, we apply both a dimensional (continuous) and categorical (binary) approach. The categorical approach applies established questionnaire cutoffs to identify individuals whose symptom levels may warrant further clinical screening. The dimensional approach assesses psychiatric symptoms across a continuum. This allows individuals to be assessed for varying levels of symptom severity, from mild or subclinical to clinically significant, without needing to meet diagnostic criteria [26, 27]. This framework also reduces the risk of missing falsely undiagnosed individuals. Combining these approaches offers a more comprehensive picture: the dimensional perspective captures variability across the full symptom range, and the categorical approach highlights potential clinical relevance. Moreover, examining different psychiatric symptom levels within the same individual allows for evaluating the relative significance of each psychiatric dimension.

We position this study as a replication-and-refinement of established chronotype-psychiatric symptom associations, extended across the adult lifespan ages, spanning a broad set of symptom domains, and estimated within a nonlinear modeling framework. This cross-sectional adult lifespan study aimed 1) to examine whether the strength or direction of associations between chronotype and psychiatric symptoms vary within adulthood (18–70 years), and 2) to map how chronotype and age relate to common psychiatric symptoms overall, partially aiming to replicate previous findings. By doing so, this study seeks to identify age-specific patterns of vulnerability and resilience associated with chronotype, thereby informing future research and guiding the development of age-tailored mental health strategies.

## Methods

### Participants

Four hundred twenty-eight participants (*M*_age_ = 45.1, *SD* = 14.0, range 18–70; 282 (65.9%) women, 143 men (33.4%), 1 non-binary, 1 queer, 1 none (0.7%)) were included in this cross-sectional analysis. Participants were recruited via an online recruitment platform (Prolific.co). The study was advertised on the Prolific website, allowing only individuals with a Prolific account to participate. Inclusion criteria were fluent in English, residing in the United Kingdom, an approval rate ≥99% in previous Prolific participations, and fitting one of the following age groups: 18–30 (*n* = 84), 31–40 (*n* = 85), 41–50 (*n* = 86), 51–60 (*n* = 93), 61–70 (*n* = 81). Recruitment aimed to stratify participants evenly across these age groups, ensuring at least 80 individuals per group. The target sample size (N ≥ 400) was set a priori to provide adequate statistical power to detect small-to-medium effects in regression models. The sample represents a convenience sample, with no strict criteria regarding chronotype or psychiatric status. Data from one participant were removed for failing more than one out of three quality checks. See Supplement for more details. There is no participant overlap between the present study and Balter et al., [25]. The study was approved by the Swedish Ethical Review Authority (dnr: 2020-03250 and 2021-01695) and carried out in accordance with the principles of the Declaration of Helsinki. Participants provided digital informed consent and were reimbursed for their time.

### Procedures and measures

All measures were completed via the participant’s personal smartphone between 11:00-17:00, completed in the order as presented below.

#### Medical diagnoses and medication intake

Self-reported medical diagnoses and medication use were collected via open-ended questions.

#### Chronotype and habitual sleep duration

The reduced Morningness-Eveningness Questionnaire (rMEQ) was used to estimate chronotype [28]. This measure consists of five questions that assess an individual’s preferred timing for activities and timing of tiredness after waking or bedtime. The total score can range between 4 and 25, with higher scores indicating greater morningness. A score of 11 or lower is indicative of being an evening chronotype and scores of 18 or higher indicate being a morning chronotype. The questionnaire has good external validity and correlates with circadian motor activity assessed using actigraphy [29]. To adjust for chronotype differences in habitual weekly sleep duration, we calculated sleep duration from self-reported bedtimes, wake times (with and without alarm use), and minutes to fall asleep on both weekdays and weekend days. Sleep duration was calculated separately for weekdays (weighted by the number of alarm versus non-alarm days) and free days (weighted by whether alarms were used). These estimates were then combined into a weighted weekly average, reflecting five weekdays and two weekend days.

#### Symptoms relating to psychiatric disorders

Validated scales of symptoms relating to 11 psychiatric disorders were completed, also including measures of symptoms that are common across disorders, such as emotion dysregulation, emotional instability, and impulsivity. Higher scores indicate greater psychiatric symptom levels. Omega Total (ω_*T*_) values as a measure of internal consistency reliability can be found in Supplement Table S1.

Depression. The Center for Epidemiologic Studies Depression Scale Revised Short Form (CESD-R 10) [30] was designed to measure depression levels in the general population, consisting of 10-items. Items are rated on a 4-point scale. The total score range is 0 to 30. A score of 10 is the suggested cutoff value for further evaluation for depression.

Generalized anxiety. The Generalized Anxiety Disorder-7 (GAD-7) [31] is a 7-item scale designed to identify probable cases of GAD. The ratings (0, 1, 2, or 3) are summed to retrieve a total score (range 0–21). The total score can be categorized as follows: minimal anxiety (0–4), mild anxiety (5–9), moderate anxiety (10–14), or severe anxiety (15–21).

Mania. The 5-item Altman Self-Rating Mania Scale (ASRM) [32] assesses the presence and severity of manic symptoms. A total score (range 0–20) is calculated by summing the item ratings (0, 1, 2, 3, 4). A score of six or higher is indicative of mania or hypomania.

Delusional ideation. The yes/no subscale of Peters Delusions Inventory 21 (PDI-21) [33] and unusual experiences subscale of the Oxford-Liverpool Inventory of Feelings and Experiences (O-LIFE) [34] were used to assess delusional ideation. The PDI-21 was designed to measure schizotypal symptom levels in the general population. The combined PDI-21 and O-LIFE unusual experiences subscale consisted of 33 yes/no items, asking for occurrence of experiences during their lifetime, such as “do you ever feel that there is a conspiracy against you?” and “are your thoughts sometimes so strong that you can almost hear them?”. Delusional ideation was scored by summing the count of all endorsements (“yes” responses) (range 0–33). There is no universally established cutoff score for the PDI-21. However, a threshold of eight endorsed items has been identified as best for discriminating individuals with a mental disorder involving psychotic features from non-clinical controls [35].

Emotion dysregulation. The Difficulties in Emotion Regulation Scale (DERS-16) [36] is a 16-item scale designed to measure emotion regulation difficulties. The total score is obtained by summing the ratings (1, 2, 3, 4, 5) of each item (range 16–80). While there is no universally established cutoff score for the DERS-16, a score of 34 or higher has been shown to best distinguish between individuals with and without depression [37].

Autism. The Autism Quotient-10 (AQ-10) [38] is a scale to measure the extent of autistic symptoms in adults. Items are rated on a 4-point scale and rescored to 0 or 1 depending on the item. Item ratings are summed to receive a total score between 0 and 10. A score of six or higher is indicative of a significant number of autistic symptoms, suitable as a rapid screen for autism spectrum disorder.

Impulsivity. The impulsivity subscale of the 20-item Health-relevant Personality Inventory (HP5i) [39] was used for this study. The four impulsivity items are rated on a 4-point scale. Item scores are averaged resulting in a score between 1 and 4. No cutoff score is available.

Emotional instability. The Affective Lability Scale (ALS-18) [40] is an 18-item scale designed to measure rapid shifts in outward emotional expressions, i.e., emotional instability. The items are rated on a 4-point scale from 0 to 3 and a total score is calculated by summing the ratings (range 0–54). There is no universally established cutoff score for the ALS-18. However, a mean score of 1.17 (average rating, rather than the sum of ratings) was observed in patients with bipolar disorder, and this is used as a tentative cutoff score [41].

Attention-Deficit Hyperactivity Disorder (ADHD). ADHD symptoms were assessed using the Adult ADHD Self-Report Scale (ASRS) [42]. The ASRS includes 18 items that measure the frequency of DSM-IV Criterion A symptoms of adult ADHD, rated on a 5-point scale: never (0), rarely (1), sometimes (2), often (3), very often (4). A total score is calculated by summing the scores (range 0–72). The “ASRS Part A”, a subset of six selected items, was used to optimize concordance with the clinical classification. For Part A, items are recoded to 0 or 1, and a score of four or more suggests symptoms levels consistent with ADHD.

Obsessive Compulsive Disorder (OCD). The Obsessive Compulsive Inventory-Revised (OCI-R) [43] is an 18-item scale to measure the severity and type of OCD symptoms present, rated on a 5-point scale. The total score is calculated by summing the item ratings (range 0–72). A score of 21 or higher suggests the presence of probable OCD.

Social anxiety. The Liebowitz Social Anxiety Scale (LSAS) [44, 45] assesses fear/anxiety and avoidance of common situations. The 24 items are rated twice. Once on fear or anxiety, rated from 0 “none” (fear or anxiety) to 3 “severe” (fear or anxiety) and once on avoidance, rated from 0 “never (0%)” to 3 “usually (67–100%)”. The total score is calculated by summing the ratings of both subscales (range 0–144). Higher scores indicate greater symptom severity. Scores are interpreted as follows: 0–29 (no social anxiety), 30–49 (mild social anxiety), 50–64 (moderate social anxiety), 65–79 (marked social anxiety), 80–94 (severe social anxiety), and 95 or above (very severe social anxiety). A score of 60 or higher is considered indicative of generalized social anxiety.

### Statistical approach

Chronotype and age associations with continuous psychiatric symptoms were assessed using fixed effects regression analysis. Predictors were Z-transformed to allow for comparison of coefficients. While primary inferences rely on continuous symptom scores, we also used the questionnaire’s established cutoff scores to assess relationships with chronotype and age. As no validated cutoff score was available for impulsivity, this variable was omitted from the categorical analyses. Logistic regression was used to examine these binary associations (below or above the cutoff score) and predicted probability curves were generated to visualize the relationships. To examine how common psychiatric symptoms develop across the adult lifespan ages and how it covaries with chronotype, generalized additive models (GAMs) were fitted using the *gam* function of the *mgcv* R package [46]. GAM is a flexible tool suitable for capturing nonlinear relationships through its use of smooth functions. In these models, both age and chronotype were treated as continuous variables with smooth functions, specified using the shrinkage version of the cubic regression spline bases, and a tensor product interaction of age and chronotype. To reduce the risk of overfitting, shrinkage smoothers were used on the predictors. This approach ensures that the smooth of a given fixed-effect predictor becomes zero if it does not meaningfully contribute to the model. Separate models were specified for each psychiatric scale. We used three-dimensional plots to visualize the relationships between three continuous variables. Prediction grid points too distant from observed data were excluded to improve the accuracy of the plotted smooth surfaces. Specifically, after rescaling predictors to a unit square, any grid nodes located more than 0.1 units from the nearest observed data point were excluded from the plot. This approach avoids overstretching extrapolations. All models were adjusted for habitual weekly sleep duration. Sleep duration was entered as a continuous predictor in the regression analyses, and as a continuous smooth term in the GAMs. We also assessed whether chronotype-age relationships differed by gender using a smooth function for age with a thin plate regression spline basis, gender as a categorical variable, and a smooth for age that was allowed to vary by gender. The effective degrees of freedom (edf) associated with each smooth term reflects the degree of nonlinearity of the smooth function [46]. An edf of 1 is equivalent to a linear relationship and a higher edf suggests a more flexible nonlinear function.

## Results

**Fig. 1:**
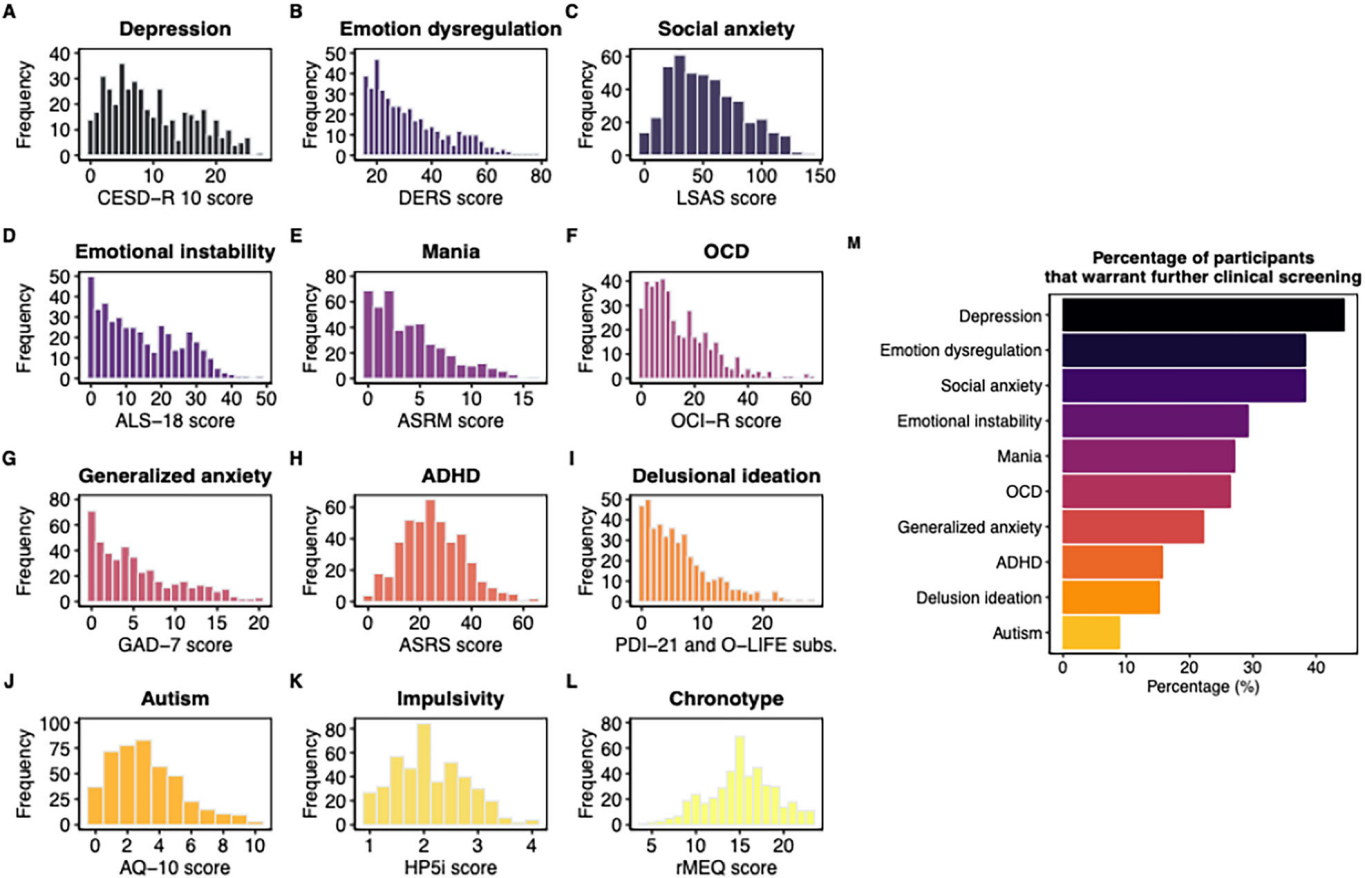
Psychiatric symptom and chronotype distributions and percentage of participants exceeding cutoffs that warrantfurther clinical screening. Frequency distributions of the (**A**–**K**) psychiatric symptom questionnaires and (**L**) chronotype scores (reduced morningness eveningness questionnaire; rMEQ); (**M**) Percentage of participants whose scores exceeded established cutoffs, indicating symptom levels that warrant further clinical screening. Impulsivity is excluded from (M) as no validated cutoff score is available.

Figure 1 presents the distribution of scores for each psychiatric symptom questionnaire (Fig. 1a) and the percentage of participants exceeding the clinical/screening cutoff for each measure (Fig. 1b). The highest proportion of individuals scoring above the cutoff was observed for symptoms relating to depression, emotion dysregulation, and social anxiety, and the lowest rate was found for autism disorder symptoms. Supplementary Table S2 provides details on the prevalence of self-reported psychiatric diagnoses (33 endorsements in total) and use of psychiatric medication (72 reports of psychotropic medication use). Notably, the cutoff-derived rates in Fig. 1b exceed the prevalence of self-reported psychiatric diagnoses. We also observe that, in several domains, psychotropic medication use is more common than self-reported psychiatric diagnoses, consistent with scenarios in which treated participants do not self-identify as having a diagnosis. Psychotropic medication use [yes/no, no serving as reference] was not significantly associated with age (b = −1.75, 95% CI [−5.49, 1.99], *p* = 0.358) or chronotype (b = −0.49, 95% CI [−1.51, 0.53], *p* = 0.347).

### Chronotype and age associations with psychiatric symptoms

#### Continuous psychiatric symptoms

**Fig. 2:**
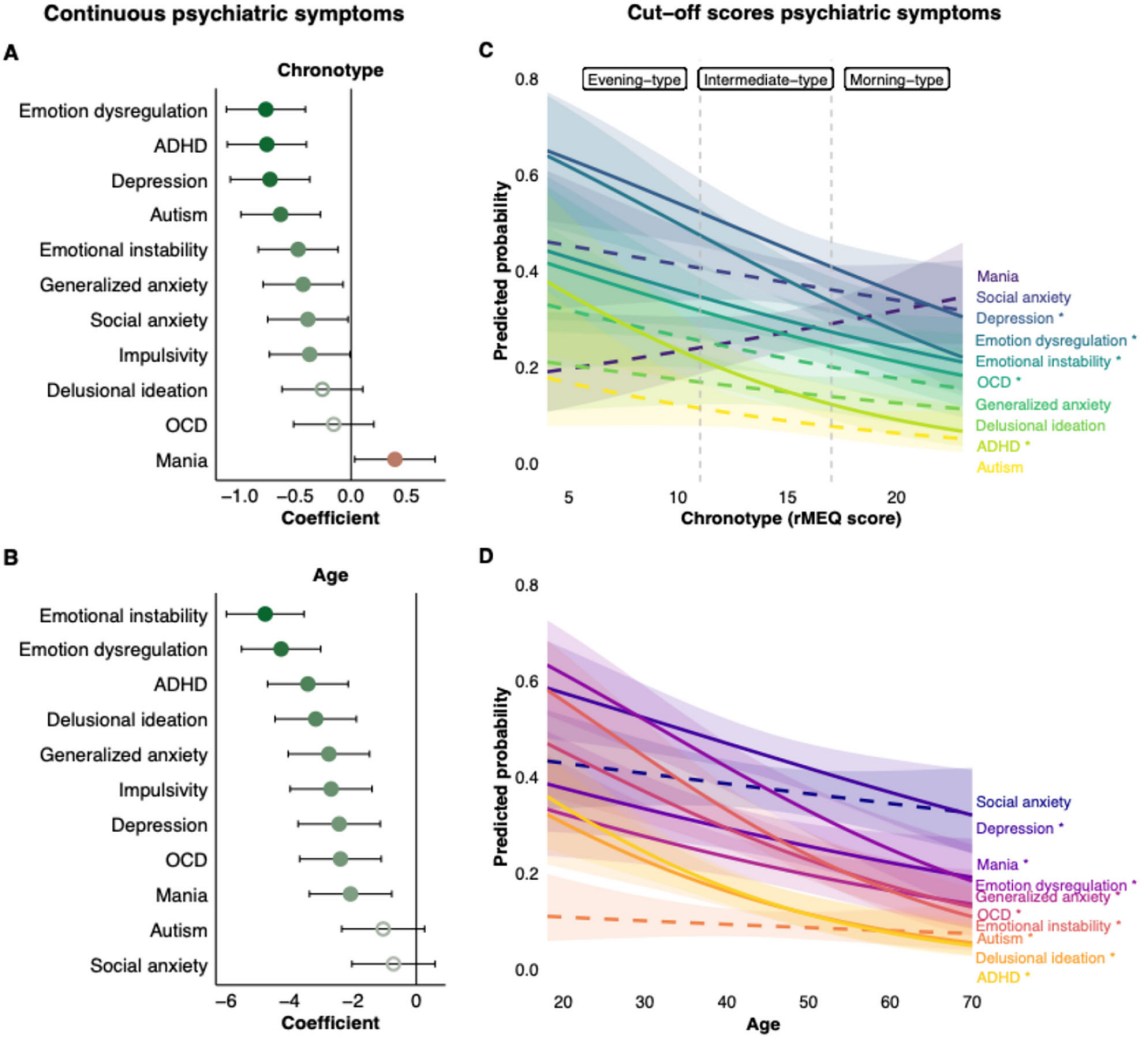
Associations of continuous and dichotomous psychiatric symptom measures with age and chronotype. **A,** **B** Coefficient plots showing the relationship between continuous psychiatric symptoms and (**A**) chronotype and (**B**) age. Symptoms are ordered by the strength of the association (0 = no relationship). Negative coefficients indicate higher symptom levels in individuals with stronger eveningness or younger age. Models are adjusted for habitual weekly sleep duration. Positive coefficients indicate higher symptom levels in individuals with stronger morningness or older age. The color intensity (darker) reflects the overall level of the coefficient. **C,** **D** Predicted probability curves showing the likelihood of scoring above the cutoff for each psychiatric questionnaire across levels of (**C**) chronotype (rMEQ) score or (**D**) age. Dashed vertical lines in panel C mark rMEQ cutoffs for chronotype categories: evening-type (≤11), intermediate-type (12–17), and morning-type ( ≥ 18). Impulsivity is not included since no clinical cutoff score is available. Error bars and ribbons represent 95% confidence intervals. Filled circles in panel A and B and solid lines and asterisks in C and D denote statistically significant associations; open circles and dashed lines indicate non-significant associations. Detailed model results can be found in Supplement Table S3–6.

*Chronotype*. As shown in Fig. 2a, eveningness (lower rMEQ scores) was associated with (in decreasing order of strength): emotion dysregulation; ADHD; depression; autism; emotional instability; generalized anxiety, social anxiety. Mania was associated with morningness (higher rMEQ scores). There were no significant associations of chronotype with delusional ideation or OCD. The smooth term for age was linearly associated with chronotype, indicating that, as expected, older age was associated with stronger morningness (higher rMEQ scores). In a linear model, older age was associated with both shorter weekly sleep duration (b = −1.81, 95% CI [−2.93, −0.69], *p* = 0.002) and stronger morningness (b = 0.74, 95% CI [0.41, 1.08], *p* < 0.001). See Supplement Figure S1 for the differing chronotype-age relationships between men and women. Individuals with other gender identities than man or woman were excluded from this analysis due to an insufficient sample size (*n* = 3). The results show that in men, but not in women, increasing age is associated with a gradual shift towards morningness. Chronotype did not significantly differ between men and women across ages (parametric coefficient: b = 0.52, t-value = 1.39, *p* = 0.166) (men serving as reference).

*Age*. As shown in Fig. 2b, psychiatric symptom levels were lower with increasing age, most strongly for emotional instability, emotion dysregulation, and ADHD, followed by delusional ideation, generalized anxiety, impulsivity, depression, OCD, and mania symptoms. Autism and social anxiety symptoms were not significantly associated with age. Supplement Figure S4 shows plots for the nonlinear equivalents, with raw scores plotted.

#### Dichotomous psychiatric symptom thresholds

*Chronotype*. We examined whether chronotype, measured continuously, was associated with the presence of clinically relevant levels of psychiatric symptoms, defined as having symptom levels above established cutoffs for each scale. As shown in Fig. 2c, greater eveningness (lower rMEQ scores) was significantly associated with increased odds of reporting clinically relevant symptoms of ADHD, emotion dysregulation, depression, OCD (note that the association with OCD was not statistically significant for continuous symptom scores), and emotional instability. No significant associations were observed for autism (note that the association with autism was statistically significant for continuous symptom scores), generalized anxiety, delusional ideation, social anxiety, and mania symptoms.

*Age*. The same analyses were conducted using age as a continuous predictor to examine its association with the presence of clinically relevant levels of psychiatric symptoms. As shown in Fig. 2d, older age was significantly associated with lower odds of meeting the cutoff for emotional instability, ADHD, delusional ideation, emotion dysregulation, OCD, generalized anxiety, depression, and mania. These findings indicate that younger individuals were more likely to report clinically relevant psychiatric symptoms across multiple psychiatric symptoms. No significant associations were observed for social anxiety or autism-related symptoms.

### Chronotype-specific changes in psychiatric symptoms across age

**Fig. 3:**
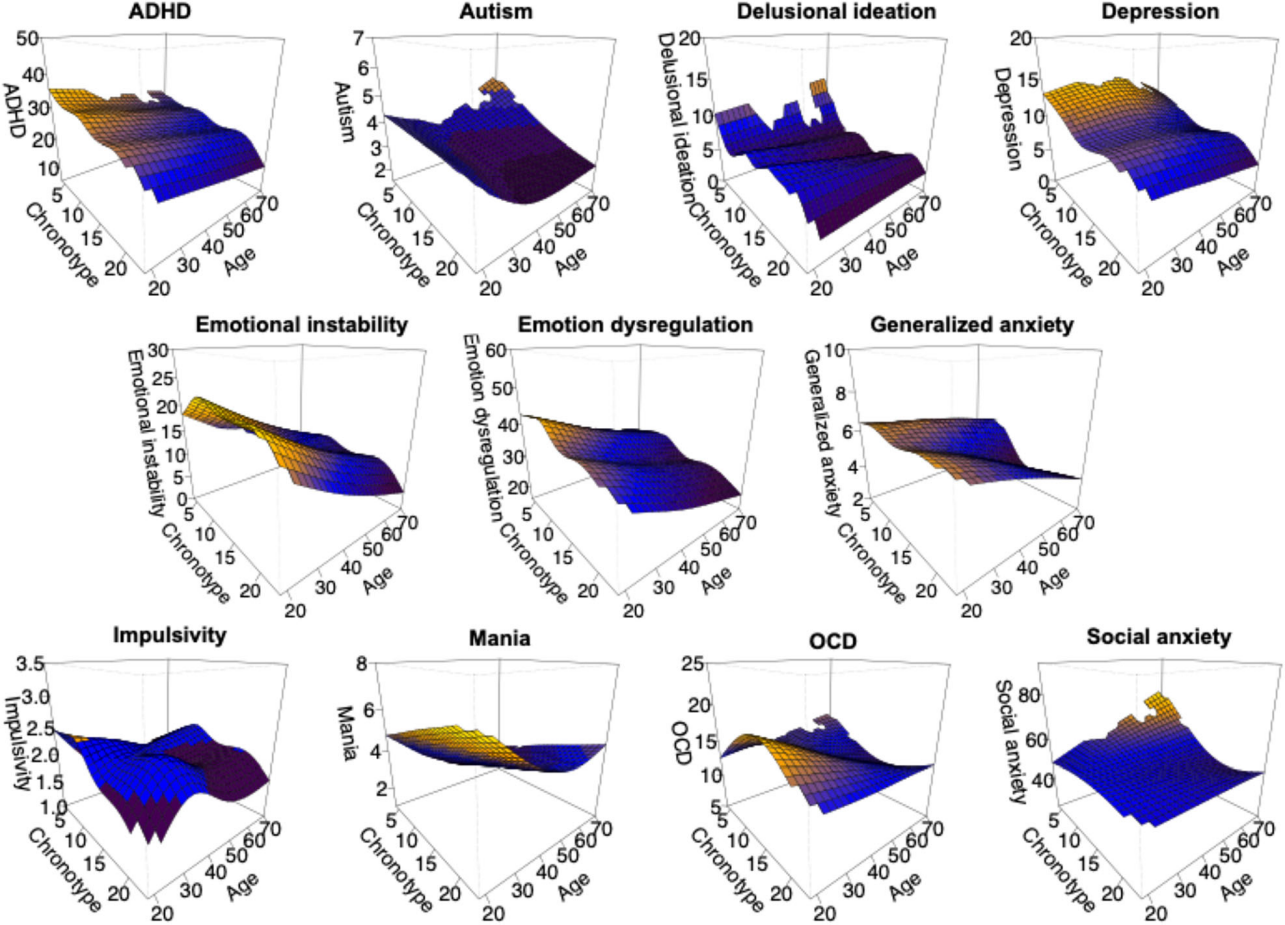
Three-dimensional plots showing predicted values of psychiatric symptoms as a function of chronotype and age. The color gradient represents the predicted intensity of the psychiatric symptoms, with warmer colors (yellow/orange) indicating higher psychiatric symptom scores on the y-axis and colder colors (blue/black) representing lower psychiatric symptom scores. Lower chronotype values reflect a tendency towards a stronger evening chronotype. The plots are based on a 26 × 26 grid, where each box represents a ~ 1-point step increase in chronotype (rMEQ) score and a 2-year increase in age. Gaps in the grid are due to nodes located more than 0.1 units from the nearest robust estimated data point are excluded (to avoid overstretching extrapolations). Models are adjusted for habitual weekly sleep duration.

Figure 3 displays the chronotype by age relationships with psychiatric symptoms across the 18–70 age span. See Supplement Figure S5 for similar plots with +/- 1.5 the standard error and Supplement Table S7 for detailed results.

With older age, symptoms were lower (emotional instability, emotion dysregulation) similarly across chronotype, or the symptom-chronotype relationship remained more stable across ages (depression, generalized anxiety, OCD, ADHD). However, among individuals with stronger eveningness, social anxiety symptoms were higher with increasing age. Delusional ideation showed a similar, though weaker, pattern. Mania was the only symptom for which individuals with stronger eveningness showed overall lower levels thantheir morningness counterparts, including at older ages. While morningness was generally associated with low or declining symptoms levels across age, autism symptoms showed a pattern with lower levels around age 45 and then gradually higher levels until age 70, which was also present in eveningness.

## Discussion

In this cross-sectional study, we examined how symptoms characteristic of common psychiatric disorders are associated with chronotype, and how these relationships differ between ages of the adult lifespan. Our findings show that psychiatric symptom levels are generally lower with increasing age, aligning with the lower prevalence of diagnostic psychiatric disorders in older adults [4]. Replicating prior (longitudinal) research, we found associations between chronotype and most psychiatric symptoms and that older individuals have a stronger tendency towards morningness. Eveningness was associated with higher levels of eight out of 11 psychiatric symptoms. One (mania symptoms) was associated with morningness. In two independent samples we found that mania was associated with morningness [25]. This is in line with acute mania being associated with an advanced circadian phase [47]. However, bipolar disorder, characterized by cyclic episodes of mania and depression separated by periods of normal affect, has been associated with eveningness [47, 48], with delayed circadian rhythms during depressive periods and normal rhythms after treatment [47, 49]. Our findings support the idea that mania and depression are differentially associated with circadian phase and highlight that studies on bipolar disorder need to distinguish between the distinct relationships manic and depressive states have with chronotype. Our study also revealed some differences from prior research. We did not find significant associations between chronotype and symptoms of OCD or delusional ideation, despite prior research linking these symptoms to eveningness [25, 50, 51] but see also [15, 50, 52]. Some of these discrepancies may be attributed to the older age range of our sample compared to previous studies, as the relationship between chronotype and psychiatric symptoms like delusional ideation and particularly social anxiety, appears to change with age. These findings highlight the importance of considering both age and chronotype as factors in psychiatry research, as these factors can influence the manifestation of symptoms across the lifespan.

Beyond treating psychiatric symptoms as continuous, we used questionnaire cutoffs to identify individuals whose symptom levels may warrant further clinical screening. These categorical analyses revealed overall consistent, although somewhat weaker, patterns with our dimensional findings, supporting that a dimensional approach is clinically relevant and can enhance power. Specifically, individuals with eveningness were more likely to exceed clinical thresholds for ADHD, emotion dysregulation, autism, depression, OCD, and emotional instability. The alignment between both approaches, lower general symptom levels and fewer individuals exceeding cutoff scores with older age, supports the idea that the lower number of diagnoses in older adults may reflect both fewer symptoms and a lower general need for clinical diagnosis with increasing age.

The changing associations between chronotype and psychiatric symptoms across the adult lifespan ages support that chronotype can play a role in the expression of psychiatric symptoms throughout adulthood, including latter life [53, 54]. In older age, levels of social anxiety were higher in those with stronger eveningness than their morningness and younger counterparts, suggesting that the combination of aging and eveningness may reflect particular vulnerability. A similar but weaker pattern was observed for delusional ideation symptoms, though delusional ideation level was not associated with eveningness when the entire age range was considered. The latter contrasts with findings from a meta-analysis of patients with schizophrenia (with a narrower age range than our study), which found that schizophrenia is related to being an evening type [55]. Future research could further explore the longitudinal relationships between age, chronotype, and different aspects of psychotic experiences, as well as other symptoms of schizophrenia. Considering that the general improvement of mental health across the lifespan largely parallels the gradual reduction in eveningness with increasing age, it is possible that becoming more of a morning type is a mediator and may contribute to some of these improvements. However, there is presently a poor understanding to what degree chronotype is a risk factor for developing psychiatric problems. Some studies report that late chronotypes have a weak but significant prospective risk of developing or experiencing a worsening of depression [56, 57], others have not found such longitudinal relationship [58], and there is also evidence that depression itself predicts future evening preference [57], and that an earlier chronotype predicted increases in depression symptoms six months later (though not 12-months later) [59]. There is clearly a need for longitudinal studies that tease out the role of chronotype, and its behavioral consequences (such as short and more irregular sleep) in the development, severity, chronicity, and recovery from psychiatric problems, and to what degree these relationships change with age. In all, the present lack of longitudinal studies leaves open the question of whether chronotype is best conceptualized as a mediator, a parallel symptom, or a consequence of psychiatric problems.

Morningness was generally associated with lower psychiatric symptom levels, with older adults who exhibited stronger morningness showing the lowest symptom levels compared to both their eveningness and younger counterparts. This is in line with the notion that morningness may offer protection against psychiatric symptoms [14]. An exception to this pattern was seen for autism symptoms, where symptoms increased from 45 years of age among individuals with stronger morningness, and mania remained consistently higher in morningness across ages. The higher symptom levels with extreme eveningness as they age warrants further investigation, as our study had a limited number of individuals in the more extreme chronotypes across the entire age spectrum. Replication in larger and stratified samples will also be important for validating and expanding upon our findings.

The demographics of many countries are changing significantly, with people aged 60 and older already outnumbering those aged five and younger in 2020. By 2030, one in six people will be aged 60 or over [60]. Currently, the prevalence of most psychiatric diagnoses is lower among older adults compared to younger groups [4, 8]. However, this trend is likely to change as many individuals diagnosed with psychiatric disorders earlier in life may carry these diagnoses into older age. While the reduced prevalence of psychiatric disorders among older adults has been attributed to factors such as changes in diagnostic practices and underdiagnosis [9], our data support that some older adults may no longer need their earlier diagnoses. The extent to which some older adults might benefit from reevaluating certain psychiatric diagnoses and potentially discontinuing medication needs further evaluation.

Our data also highlight a need for vigilance in identifying older adults who may be vulnerable to specific psychiatric disorders or symptoms, such as social anxiety among individuals with stronger eveningness, and mania symptoms among those with morningness. This calls for tailored approaches to mental health care that consider both age-related changes and chronotype.

The primary limitation of the present study is its cross-sectional design, which does not capture intraindividual variation in trajectories and restricts the analysis to interindividual differences. Future research may consider longitudinal or multicohort longitudinal designs. Additionally, the use of a convenience sample limits the generalizability of findings, particularly to extreme chronotypes. The finding that more extreme chronotypes are more likely to suffer from specific psychiatric symptoms requires replication in stratified samples with a larger number of extreme chronotypes. Future research may also assess the mechanisms underlying the chronotype-psychiatric symptom relationships, investigate how these mechanisms may differ between younger and older age groups, and assess the role of symptom duration/chronicity (an aspect that was not captured by our symptom measures). The importance of other factors that can explain differences between chronotypes, such as personality (where e.g., extraversion and openness have been linked to eveningness [61]), may also be fruitful to explore further. Further, we capped data collection at the age of 70. Future work may also test the oldest-old adults, as well as children and adolescents to capture the full lifespan spectrum. This focus on an adult-only sample means that the patterns likely index post-onset changes in some psychiatric symptoms, not initial onset processes. Although self-reports of psychiatric illness are generally good indicators of actual psychiatric illness [62], our findings show discrepancies between self-reported diagnoses, medication intake, and cutoff-derived rates from questionnaires. These may reflect a combination of (i) subthreshold symptomatology or underdiagnosis, (ii) a person may once have had a diagnosis but currently may not meet symptom thresholds, and/or (iii) psychiatric medication use without endorsing a diagnosis (e.g., past diagnosis not recalled, label avoidance, or off-label use). Accordingly, questionnaire cutoff categories do not provide a perfect proxy for clinical diagnosis, and categorical analyses should be interpreted with these constraints in mind. Lastly, while remote testing methods (such as smartphone-based testing or online platforms) offer advantages such as broad participant reach and reduced logistical, physical, and psychological barriers to participation in research, a limitation is the reduced control over participants’ testing environments. Factors such as background noise, distractions, and potential technological issues can introduce variability and potentially affect data quality, beyond what is captured by quality checks. Nonetheless, the Prolific platform has consistently been rated as the top platform for data quality when compared to alternatives [63, 64]. Furthermore, the data used here were based solely on questionnaire data, which are generally less sensitive to environmental variability than performance-based tasks. Replication of our results across different populations, study designs, and methods of data collection is necessary to further establish their robustness.

Strengths of this study include the comprehensive assessment of psychiatric symptoms within individuals, allowing for a broad understanding of how the relationships between psychiatric symptoms and chronotype change across adult lifespan ages. This approach also allows for evaluating the significance of each psychiatric dimension relative to each other. By focusing on psychiatric symptom levels rather than formal psychiatric diagnoses, the study aligns with dimensional approaches such as the Research Domain Criteria (RDoC). This framework aims to improve our understanding of mental ill-health by studying how common disruptions in psychological and neurobiological systems contribute to psychiatric symptoms across traditional diagnostic boundaries [65]. Thus, a symptom level focus can help clarify the mechanisms underlying variations in symptom severity in the general population, such as a person’s chronotype in this study, but may not directly translate to specific patient groups.

In summary, as the population ages, it becomes increasingly important to adapt psychiatric diagnostics and interventions to better reflect the evolving needs and characteristics of older adults. This includes improving the detection of mental health issues that may emerge, persist, or decline in later life. Our findings support that psychiatric symptoms are generally lower in older adults, but there may be specific vulnerabilities relating to both age and chronotype. For example, emotion-related psychiatric symptoms such as emotional instability and emotion dysregulation, were most pronounced in younger adults with eveningness, whereas social anxiety symptoms peaked in older adults with eveningness. Conversely, morningness was generally linked to lower levels of psychiatric symptoms, particularly in older age, though mania symptoms were higher in younger and older adults with morningness compared to those with eveningness.

## Supplementary information

SUPPLEMENTAL MATERIAL

## Data Availability

Analysis code is available upon request.
