# Supplementary material for: Associations between chronotype and psychiatric symptoms across the adult lifespan
Source: Transl Psychiatry. 2025 Dec 5;15:522. doi: 10.1038/s41398-025-03782-w (PMC12689609; doi:10.1038/s41398-025-03782-w)
Supplement: Supplementary file 1 — SUPPLEMENTAL MATERIAL [file 41398_2025_3782_MOESM1_ESM.docx]

**SUPPLEMENTARY MATERIAL**

**The Associations Between Chronotype and Psychiatric Dimensions Change With Age**

Axelsson & Balter

Table of Contents

[SUPPLEMENTARY FIGURES 3](#_Toc213255415)

[**Figure S1.** The association between chronotype and age as a function of gender. 3](#_Toc213255416)

[**Figure S2.** Frequency plot of gender by age category. 4](#_Toc213255417)

[**Figure S3.** Histogram of habitual weekly sleep duration. 5](#_Toc213255418)

[**Figure S4.** Relationships between psychiatric symptom scores and age. 6](#_Toc213255419)

[**Figure S5.** Three-dimensional plots of predicted values of psychiatric symptoms as a function of chronotype and age. 7](#_Toc213255420)

[SUPPLEMENTARY TABLES 8](#_Toc213255421)

[**Table S1.** Internal consistency reliability assessed using Omega Total (ωT) for each questionnaire. 8](#_Toc213255422)

[**Table S2**. Self-reported medical diagnoses and medication intake by age category. 9](#_Toc213255423)

[**Table S3.** Fixed effects regression analysis results of the relationship between chronotype and psychiatric symptoms, adjusted for habitual weekly sleep duration. 10](#_Toc213255424)

[**Table S4.** Fixed effects regression analysis results of the relationship between age and psychiatric symptoms, adjusted for habitual weekly sleep duration. 11](#_Toc213255425)

[**Table S5.** Results from generalized linear models examining the association between chronotype and the presence of psychiatric symptoms 12](#_Toc213255426)

[**Table S6.** Results from generalized linear models examining the association between age and the presence of psychiatric symptoms 13](#_Toc213255427)

[**Table S7.** GAM results of the age-chronotype relationships for each psychiatric symptom questionnaire, adjusted for habitual weekly sleep duration. 14](#_Toc213255428)

#
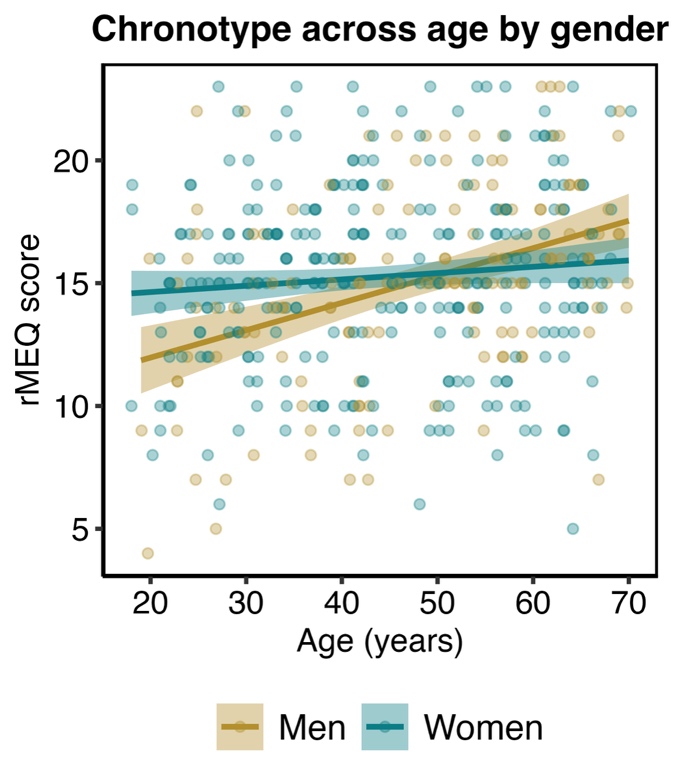
SUPPLEMENTARY FIGURES

**Figure S1.** The association between chronotype and age as a function of gender. **Gender identities other than men (n = 143) or women (n = 282) were excluded (n = 3) because the number of data points was deemed insufficient for valid analysis on gender. A lower reduced Morningness-Eveningness Questionnaire (rMEQ) score indicates stronger eveningness. The results show that in men (but not in women), older age is associated with a gradual shift towards morningness. Analyses were conducted using generalized additive models, which allow for nonlinear relationships. See Supplementary Figure S2 for a** frequency plot of gender by age category.

## **Figure S2.** Frequency plot of gender by age category.

## **Figure S3.** Histogram of habitual weekly sleep duration. Mean = 7h28, SD = 1h08.

**
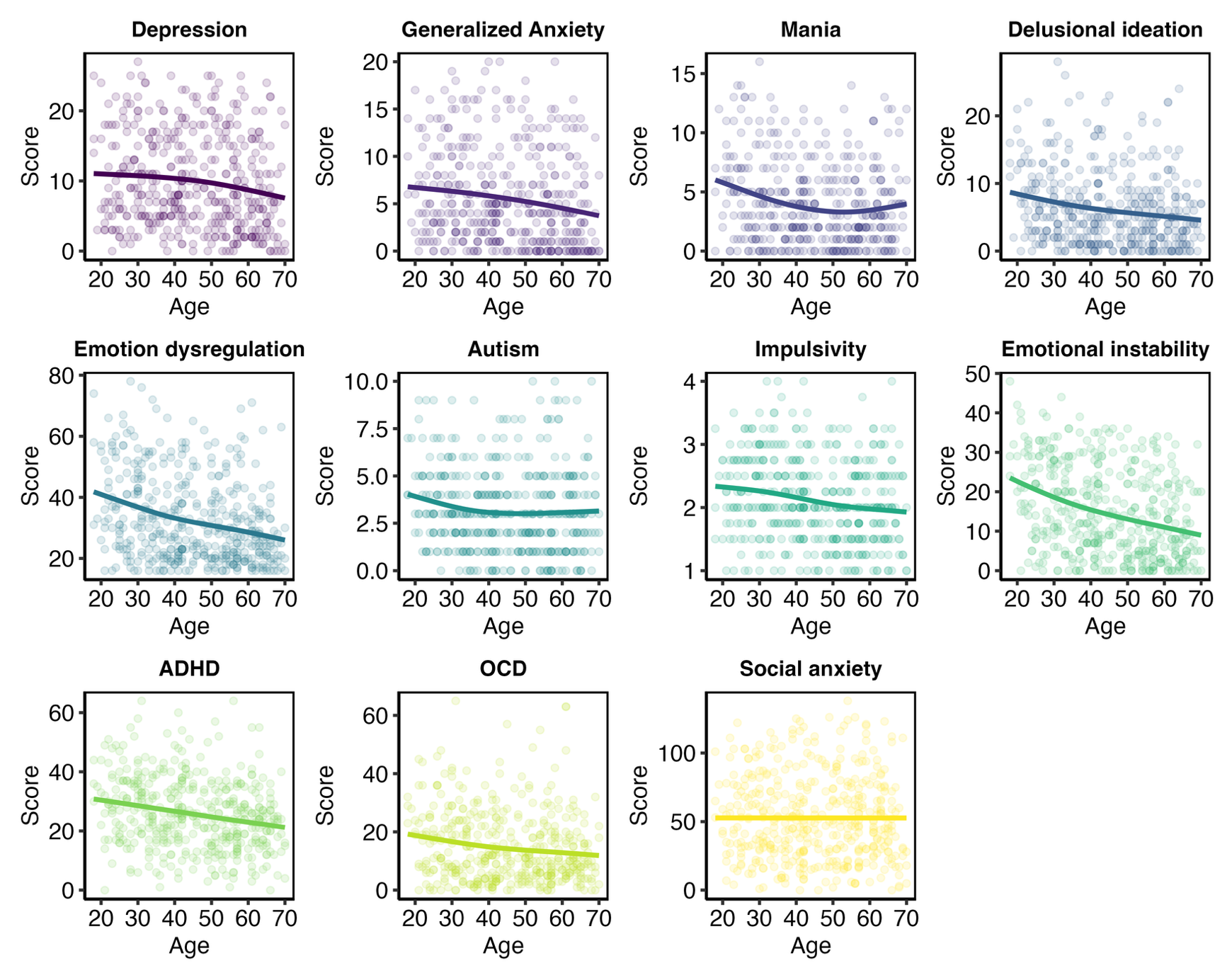
****Figure S4.** Relationships between psychiatric symptom scores and age. **Plots display raw values with a penalized regression spline smooth.** Models are adjusted for habitual weekly sleep duration.

**Figure S5.** Three-dimensional plots of predicted values of psychiatric symptoms as a function of chronotype and age. **The red and green layers represent +/- 1.5 standard errors, respectively. The black layers represent the predicted values (shown in Figure 2). A lower rMEQ score (reduced Morningness-Eveningness Questionnaire) indicates stronger eveningness. The plots are based on a 26 x 26 grid, where each box represents a ~1- point step increase in chronotype (rMEQ)** score and a 2-year increase in age. Models are adjusted for habitual weekly sleep duration.

# SUPPLEMENTARY TABLES

## **Table S1. Internal consistency reliability assessed using Omega Total (ωT) for each questionnaire.**

| **Questionnaire** | ω*_T_* |
| --- | --- |
| Depression (CESD-R 10) | 0.91 |
| Generalized anxiety (GAD-7) | 0.91 |
| Mania (ASRM) | 0.79 |
| Delusional ideation (PDI-21 and O-LIFE subscale) | 0.88 |
| Emotion dysregulation (DERS-16) | 0.97 |
| Autism (AQ-10) | 0.67 |
| Impulsivity (HP5i subscale) | 0.76 |
| Emotional instability (ALS-18) | 0.95 |
| ADHD (ASRS) | 0.92 |
| OCD (OCI-R) | 0.93 |
| Social anxiety (LSAS) | 0.98 |
| Chronotype (rMEQ) | 0.72 |

**Table S2**. Self-reported medical diagnoses and medication intake by age category. **Values represent count and percentage from total N in age group.**

|  | **18-30**  ***n* = 84** | **31-40**  ***n* = 85** | **41-50**  ***n* = 86** | **51-60**  ***n* = 93** | **61-70**  ***n* = 81** |
| --- | --- | --- | --- | --- | --- |
| **Self-reported psychiatric diagnosis, *n* (%)** | 10  (11.9%) | 5  (6.0%) | 5  (5.8%) | 8  (8.6%) | 5  (6.2%) |
| Depressive disorder, *n* (%) | 3  (3.6%) | 1  (1.2%) | 1  (1.2%) | 3  (3.2%) | 1  (1.2%) |
| Anxiety disorder, *n* (%)  (GAD, panic disorder) | 3  (3.6%) | 4  (4.8%) | 4  (4.7%) | 5  (5.4%) | 2  (2.5%) |
| Compulsive disorder, *n* (%) | 2  (2.4%) | 0 | 0 | 0 | 0 |
| Personality disorder, *n* (%) | 1  (1.2%) | 0 | 0 | 0 | 0 |
| Post-Traumatic Stress  Disorder, *n* (%) | 1  (1.2%) | 0 | 0 | 0 | 0 |
| ADHD or autism, *n* (%) | 0 | 0 | 0 | 0 | 2  (2.5%) |
| **Psychotropic medication, *n* (%)** |  |  |  |  |  |
| Anti-depressant/anxiety, *n* (%) | 12  (14.3%) | 17  (20.2%) | 14  (16.3%) | 11  (11.8%) | 8  (9.9%) |
| Anti-psychotic, *n* (%) | 1  (1.2%) | 0 | 0 | 0 | 0 |
| Mood stabilizer, *n* (%) | 0 | 1  (1.2%) | 1  (1.2%) | 1  (1.1%) | 0 |
| Pain medication, n (%) | 0 | 2  (2.4%) | 1  (1.2%) | 2  (2.2%) | 1  (1.2%) |
| **Other medication (non-**  **psychiatric), *n* (%)** | 4  (4.8%) | 7 (8.3%) | 10  (11.6%) | 12  (12.9%) | 26  (32.1%) |

*Note.* GAD = Generalized Anxiety Disorder; ADHD = Attention Deficit Hyperactivity Disorder. Anti-depressant/anxiety medication include selective serotonin reuptake inhibitors (SSRIs), serotonin-norepinephrine reuptake inhibitors (SNRIs), tricyclic antidepressants (TCAs), atypical antidepressants, benzodiazepines, and anxiolytic (non-benzodiazepine); Other medication (non-psychiatric) include e.g., cardiovascular, respiratory, gastrointestinal, diabetes, thyroid, and migraine medication.

**Table S3.** Fixed effects regression analysis results of the relationship between chronotype and psychiatric symptoms, adjusted for habitual weekly sleep duration. **Results correspond to the results visualized in Figure 2a in the main text. Predictors were Z-transformed to allow for comparison of coefficients. For example, a one standard deviation increase in emotion dysregulation was associated with a 0.77-point decrease on the chronotype scale.**

**Chronotype and continuous psychiatric symptoms**

| **Model** | **Coefficient** | **95% confidence interval** | ***p*-value** |
| --- | --- | --- | --- |
| Emotion dysregulation | **-0.77** | **-1.12, -0.41** | **0.000** |
| *Sleep duration* | 0.09 | -0.27, 0.44 | 0.626 |
| ADHD | **-0.76** | **-1.11, -0.40** | **0.000** |
| *Sleep duration* | 0.07 | -0.29, 0.42 | 0.701 |
| Depression | **-0.73** | **-1.08, -0.37** | **0.000** |
| *Sleep duration* | 0.04 | -0.32, 0.40 | 0.835 |
| Autism | **-0.63** | **-0.99, -0.28** | **0.001** |
| *Sleep duration* | 0.18 | -0.18, 0.54 | 0.322 |
| Emotional instability | **-0.48** | **-0.83, -0.12** | **0.009** |
| *Sleep duration* | 0.11 | -0.24, 0.47 | 0.532 |
| Generalized anxiety | **-0.43** | **-0.79, -0.07** | **0.019** |
| *Sleep duration* | 0.10 | -0.26, 0.46 | 0.598 |
| Social anxiety | **-0.39** | **-0.75, -0.03** | **0.035** |
| *Sleep duration* | 0.10 | -0.26, 0.46 | 0.583 |
| Impulsivity | **-0.37** | **-0.73, -0.01** | **0.044** |
| *Sleep duration* | 0.14 | -0.22, 0.50 | 0.443 |
| Delusional ideation | -0.26 | -0.62, 0.10 | 0.163 |
| *Sleep duration* | 0.10 | -0.26, 0.46 | 0.587 |
| OCD | -0.16 | -0.52, 0.20 | 0.395 |
| *Sleep duration* | 0.13 | -0.23, 0.49 | 0.473 |
| Mania | **0.39** | **0.03, 0.75** | **0.033** |
| *Sleep duration* | 0.08 | -0.28, 0.45 | 0.650 |

*Note.* Bold values denote *p* < .05.

**Table S4.** Fixed effects regression analysis results of the relationship between age and psychiatric symptoms, adjusted for habitual weekly sleep duration. **Results correspond to the results visualized in Figure 2b in the main text. Predictors were Z-transformed to allow for comparison of coefficients. For example, a one standard deviation increase in emotional instability was associated with being 4.77 years younger.**

**Age and continuous psychiatric symptoms**

| **Model** | **Coefficient** | **95% confidence interval** | ***p*-value** |
| --- | --- | --- | --- |
| Emotional instability | **-4.77** | **-6.00, -3.55** | **0.000** |
| *Sleep duration* | **-2.15** | **-3.38, -0.93** | **0.001** |
| Emotion dysregulation | **-4.27** | **-5.52, -3.02** | **0.000** |
| *Sleep duration* | **-2.22** | **-3.47, -0.97** | **0.001** |
| ADHD | **-3.43** | **-4.70, -2.15** | **0.000** |
| *Sleep duration* | **-2.25** | **-3.53, -0.98** | **0.001** |
| Delusional ideation | **-3.18** | **-4.46, -1.89** | **0.000** |
| *Sleep duration* | **-2.37** | **-3.65, -1.08** | **0.000** |
| Generalized anxiety | **-2.76** | **-4.05, -1.48** | **0.000** |
| *Sleep duration* | **-2.20** | **-3.49, -0.91** | **0.001** |
| Impulsivity | **-2.69** | **-3.98, -1.40** | **0.000** |
| *Sleep duration* | **-1.91** | **-3.20, -0.63** | **0.004** |
| Depression | **-2.44** | **-3.73, -1.14** | **0.000** |
| *Sleep duration* | **-2.28** | **-3.58, -0.99** | **0.001** |
| OCD | **-2.40** | **-3.68, -1.11** | **0.000** |
| *Sleep duration* | **-1.98** | **-3.27, -0.69** | **0.003** |
| Mania | **-2.08** | **-3.38, -0.77** | **0.002** |
| *Sleep duration* | **-1.71** | **-3.01, -0.40** | **0.010** |
| Autism | -1.05 | -2.35, 0.26 | 0.117 |
| *Sleep duration* | **-1.89** | **-3.20, -0.58** | **0.005** |
| Social anxiety | -0.72 | -2.03, 0.59 | 0.282 |
| *Sleep duration* | **-2.03** | **-3.34, -0.72** | **0.003** |

*Note.* Bold values denote *p* < .05.

**Table S5.** Results from generalized linear models examining the association between chronotype and the presence of psychiatric symptoms**, defined dichotomously (yes/no) based on established questionnaire cut-offs,** adjusted for habitual weekly sleep duration**. These results correspond to the visualizations presented in Figure 2c of the main text.** For example, each additional chronotype point was associated with a 11% lower likelihood of meeting the cutoff for ADHD, after adjusting for habitual weekly sleep duration.

**Chronotype and categorical psychiatric symptoms**

| **Model** | **Odds ratio** | **95% confidence interval** | ***p*-value** |
| --- | --- | --- | --- |
| ADHD | **0.89** | **0.83, 0.96** | **0.001** |
| *Sleep duration* | 0.96 | 0.77, 1.20 | 0.724 |
| Emotion dysregulation | **0.91** | **0.86, 0.96** | **0.000** |
| *Sleep duration* | 0.99 | 0.83, 1.17 | 0.886 |
| Autism | 0.93 | 0.85, 1.01 | 0.094 |
| *Sleep duration* | 1.16 | 0.88, 1.55 | 0.302 |
| Depression | **0.93** | **0.88, 0.97** | **0.004** |
| *Sleep duration* | **0.81** | **0.69, 0.96** | **0.018** |
| OCD | **0.94** | **0.89, 0.99** | **0.032** |
| *Sleep duration* | 1.04 | 0.86, 1.25 | 0.682 |
| Emotional instability | **0.94** | **0.89, 1.00** | **0.039** |
| *Sleep duration* | 0.90 | 0.75, 1.08 | 0.251 |
| Generalized anxiety | 0.95 | 0.89, 1.01 | 0.087 |
| *Sleep duration* | 0.87 | 0.71, 1.06 | 0.159 |
| Delusional ideation | 0.96 | 0.90, 1.03 | 0.263 |
| *Sleep duration* | **0.77** | **0.61, 0.96** | **0.023** |
| Social anxiety | 0.97 | 0.92, 1.02 | 0.221 |
| *Sleep duration* | 0.90 | 0.76, 1.07 | 0.222 |
| Mania | 1.04 | 0.99, 1.10 | 0.136 |
| *Sleep duration* | 1.13 | 0.94, 1.37 | 0.212 |

*Note.* Bold values denote *p* < .05.

**Table S6.** Results from generalized linear models examining the association between age and the presence of psychiatric symptoms**, defined dichotomously (yes/no) based on established questionnaire cut-offs, adjusted for** habitual weekly sleep duration**. These results correspond to the visualizations presented in Figure 2d of the main text.** For example, each additional year of age was associated with a 5% lower likelihood of meeting the cutoff for emotional instability, after adjusting for habitual weekly sleep duration.

**Age and categorical psychiatric symptoms**

| **Model** | **Odds ratio** | **95% confidence interval** | ***p*-value** |
| --- | --- | --- | --- |
| Emotional instability | **0.95** | **0.94, 0.97** | **0.000** |
| *Sleep duration* | **0.81** | **0.66, 0.98** | **0.029** |
| ADHD | **0.95** | **0.93, 0.97** | **0.000** |
| *Sleep duration* | 0.86 | 0.68, 1.08 | 0.198 |
| Delusional ideation | **0.96** | **0.94, 0.98** | **0.000** |
| *Sleep duration* | **0.70** | **0.55, 0.88** | **0.003** |
| Emotion dysregulation | **0.96** | **0.95, 0.98** | **0.000** |
| *Sleep duration* | 0.91 | 0.76, 1.08 | 0.274 |
| OCD | **0.97** | **0.95, 0.98** | **0.000** |
| *Sleep duration* | 0.97 | 0.80, 1.17 | 0.734 |
| Generalized anxiety | **0.98** | **0.96, 0.99** | **0.010** |
| *Sleep duration* | 0.82 | 0.67, 1.01 | 0.061 |
| Depression | **0.98** | **0.97, 0.99** | **0.004** |
| *Sleep duration* | **0.78** | **0.65, 0.92** | **0.004** |
| Mania | **0.98** | **0.97, 1.00** | **0.016** |
| *Sleep duration* | 1.09 | 0.91, 1.32 | 0.358 |
| Social anxiety | 0.99 | 0.98, 1.01 | 0.217 |
| *Sleep duration* | 0.88 | 0.74, 1.05 | 0.153 |
| Autism | 0.99 | 0.97, 1.02 | 0.484 |
| *Sleep duration* | 1.14 | 0.85, 1.54 | 0.389 |

*Note.* Bold values denote *p* < .05.

**Table S7.** GAM results of the age-chronotype relationships for each psychiatric symptom questionnaire, adjusted for habitual weekly sleep duration. **These results correspond to the visualizations presented in Figure 3 of the main text.**

| **ADHD** | | | | |
| --- | --- | --- | --- | --- |
| Parametric coefficients | Estimate | SE | t-value | *p*-value |
| Intercept | **25.71** | **0.54** | **47.27** | **0.000** |
|  |  |  |  |  |
| Smooth terms | EDF | RefDF | F-value | *p*-value |
| Age | **1.26** | **7** | **2.54** | **0.000** |
| Chronotype | **4.64** | **9** | **1.96** | **0.001** |
| Sleep duration | **0.94** | **9** | **0.54** | **0.017** |
| Age x chronotype | 0.00 | 24 | 0.00 | 0.480 |
| **Autism** |  |  |  |  |
| Parametric coefficients | Estimate | SE | t-value | *p*-value |
| Intercept | **3.20** | **0.10** | **30.56** | **0.000** |
|  |  |  |  |  |
| Smooth terms | EDF | RefDF | F-value | *p*-value |
| Age | **2.40** | **9** | **0.67** | **0.009** |
| Chronotype | 0.00 | 9 | 0.00 | 0.063 |
| Sleep duration | 0.35 | 9 | 0.06 | 0.213 |
| Age x chronotype | **2.11** | **24** | **0.41** | **0.002** |
| **Delusional ideation** | | | | |
| Parametric coefficients | Estimate | SE | t-value | *p*-value |
| Intercept | **6.17** | **0.25** | **25.13** | **0.000** |
|  |  |  |  |  |
| Smooth terms | EDF | RefDF | F-value | *p*-value |
| Age | **0.00** | **9** | **0.00** | **0.002** |
| Chronotype | **6.51** | **9** | **1.04** | **0.046** |
| Sleep duration | **3.61** | **9** | **3.21** | **0.000** |
| Age x chronotype | **3.21** | **24** | **0.96** | **0.000** |
| **Depression** |  |  |  |  |
| Parametric coefficients | Estimate | SE | t-value | *p*-value |
| Intercept | **9.79** | **0.30** | **32.21** | **0.000** |
|  |  |  |  |  |
| Smooth terms | EDF | RefDF | F-value | *p*-value |
| Age | **1.28** | **9** | **0.70** | **0.001** |
| Chronotype | **3.77** | **9** | **1.20** | **0.007** |
| Sleep duration | **3.00** | **9** | **2.06** | **0.000** |
| Age x chronotype | **1.19** | **24** | **0.07** | **0.170** |
| **Emotional instability** | | | | |
| Parametric coefficients | Estimate | SE | t-value | *p*-value |
| Intercept | **14.66** | **0.50** | **29.61** | **0.000** |
|  |  |  |  |  |
| Smooth terms | EDF | RefDF | F-value | *p*-value |
| Age | **1.76** | **7** | **7.52** | **0.000** |
| Chronotype | 4.14 | 9 | 0.71 | 0.164 |
| Sleep duration | **2.93** | **9** | **1.29** | **0.004** |
| Age x chronotype | 0.00 | 24 | 0.00 | 0.449 |
| **Emotion dysregulation** |  |  |  |  |
| Parametric coefficients | Estimate | SE | t-value | *p*-value |
| Intercept | **32.46** | **0.62** | **52.31** | **0.000** |
|  |  |  |  |  |
| Smooth terms | EDF | RefDF | F-value | *p*-value |
| Age | **1.72** | **9** | **3.97** | **0.000** |
| Chronotype | **4.12** | **9** | **1.46** | **0.007** |
| Sleep duration | **2.14** | **9** | **0.82** | **0.018** |
| Age x chronotype | 0.00 | 24 | 0.00 | 0.623 |
| **Generalized anxiety** | | | | |
| Parametric coefficients | Estimate | SE | t-value | *p*-value |
| Intercept | **5.44** | **0.23** | **23.64** | **0.000** |
|  |  |  |  |  |
| Smooth terms | EDF | RefDF | F-value | *p*-value |
| Age | **1.09** | **9** | **1.08** | **0.000** |
| Chronotype | 0.00 | 9 | 0.00 | 0.279 |
| Sleep duration | **2.21** | **9** | **0.96** | **0.010** |
| Age x chronotype | 2.33 | 24 | 0.22 | 0.051 |
| **Impulsivity** |  |  |  |  |
| Parametric coefficients | Estimate | SE | t-value | *p*-value |
| Intercept | **2.11** | **0.03** | **69.85** | **0.000** |
|  |  |  |  |  |
| Smooth terms | EDF | RefDF | F-value | *p*-value |
| Age | 0.00 | 9 | 0.00 | 0.425 |
| Chronotype | **4.54** | **9** | **0.97** | **0.020** |
| Sleep duration | 3.28 | 9 | 0.55 | 0.181 |
| Age x chronotype | **6.49** | **24** | **1.17** | **0.000** |
| **Mania** | | | | |
| Parametric coefficients | Estimate | SE | t-value | *p*-value |
| Intercept | **3.95** | **0.16** | **24.60** | **0.000** |
|  |  |  |  |  |
| Smooth terms | EDF | RefDF | F-value | *p*-value |
| Age | **2.22** | **9** | **2.21** | **0.000** |
| Chronotype | **1.24** | **9** | **0.86** | **0.003** |
| Sleep duration | 2.98 | 9 | 0.65 | 0.094 |
| Age x chronotype | 0.00 | 24 | 0.00 | 0.506 |
| **OCD** |  |  |  |  |
| Parametric coefficients | Estimate | SE | t-value | *p*-value |
| Intercept | **14.70** | **0.56** | **26.21** | **0.000** |
|  |  |  |  |  |
| Smooth terms | EDF | RefDF | F-value | *p*-value |
| Age | **0.00** | **7** | **0.00** | **0.029** |
| Chronotype | 0.00 | 9 | 0.00 | 0.833 |
| Sleep duration | 2.67 | 9 | 0.51 | 0.142 |
| Age x chronotype | **4.02** | **24** | **0.61** | **0.001** |
| **Social anxiety** | | | | |
| Parametric coefficients | Estimate | SE | t-value | *p*-value |
| Intercept | **52.51** | **1.44** | **36.39** | **0.000** |
|  |  |  |  |  |
| Smooth terms | EDF | RefDF | F-value | *p*-value |
| Age | 0.00 | 9 | 0.00 | 0.779 |
| Chronotype | 0.00 | 9 | 0.00 | 0.425 |
| Sleep duration | **2.31** | **9** | **1.10** | **0.006** |
| Age x chronotype | **3.47** | **24** | **0.34** | **0.030** |

*Note*. EDF = effective degrees of freedom; RefDF = reference degrees of freedom. *Note.* Bold values denote *p* < .05.
